# Visiting Lists for 1884

**Published:** 1883-12

**Authors:** 


VISITING LISTS.—We desire to acknowledge the receipt of a copy of "The Physicians Visiting List" for 1884, from the well known publishers of Medical Works, P. Blackiston Son & Co., of Philadelphia. For many years we have been using this "Visiting List" as a "dental appointment book," and the present edition for the coming year is so perfectly arranged, that besides the useful information it contains, it answers admirably for the dentist as well as for the physician.

We are also in receipt of the "Physiciana Daily Pocket Record," a visiting list first published by the late Dr. S. W. Butler, but was edited by Dr. D. G. Brinton, Editor of the *Medical and Surgical Reporter*, Philadelphia, and from the office of which it is issued. It contains many useful memoranda, tables etc., and is well adapted to the purpose for which it is published.

We have also received a copy of "The Medical Record Visiting List and Physicians Diary," for 1884, from the well known publishers, William Wood & Co., New York, which also contains valuable information for the practicing physician with special memoranda and space for sixty patients per week.

